# Temporospatial guidance of activity-dependent gene expression by microRNA: mechanisms and functional implications for neural plasticity

**DOI:** 10.1093/nar/gky1235

**Published:** 2018-12-07

**Authors:** Dylan Kiltschewskij, Murray J Cairns

**Affiliations:** 1 School of Biomedical Sciences and Pharmacy, The University of Newcastle, Callaghan, NSW, 2323, Australia; 2 Centre for Brain and Mental Health Research, Hunter Medical Research Institute, New Lambton, NSW, 2323, Australia

## Abstract

MicroRNA are major regulators of neuronal gene expression at the post-transcriptional and translational levels. This layer of control is critical for spatially and temporally restricted gene expression, facilitating highly dynamic changes to cellular structure and function associated with neural plasticity. Investigation of microRNA function in the neural system, however, is at an early stage, and many aspects of the mechanisms employing these small non-coding RNAs remain unclear. In this article, we critically review current knowledge pertaining to microRNA function in neural activity, with emphasis on mechanisms of microRNA repression, their subcellular remodelling and functional impacts on neural plasticity and behavioural phenotypes.

## INTRODUCTION

Neurons are characterized by their ability to rapidly integrate, store and transmit synaptic stimuli received from a multiplicity of sources. This complex nature is thought to be underpinned by the inherent plasticity of neuronal structure and excitability, both of which are intrinsically linked and associated with modulation of neuronal communication and information storage in complex organisms (1,2). In recent years, it has become increasingly clear that distinct patterns of gene expression are responsible for regulating the duration and strength of plastic modifications to the neuron. For instance, post-translational modification, trafficking and degradation of synapse-associated proteins are thought to regulate short-term forms of synaptic plasticity (3). In contrast, longer-lasting forms of overall neuronal plasticity—such as long-term potentiation (LTP) and long-term depression (LTD)—generally rely on a comparably deeper response involving mRNA translation, post-transcriptional regulation, mRNA transcription and epigenetic DNA modifications (3).

The regulation of mRNA translation, in particular, has been subject to increasing focus due to its intricate nature and extensive potential for fine-tuning of neuronal remodelling. Since the discovery of active ribosomes in dendritic spines (4,5), it has become increasingly clear that translation is decentralised in neurons, likely to allow various subcellular compartments to express proteins independently and in a rapid manner at the site of need. In support of this more than 2,500 unique mRNAs were detected in the neurites of hippocampal neurons (6), suggesting distal regions of the neuron possess highly mobile and specific transcriptomes. Recent evidence also suggests that these transcripts are highly active in synaptic activity and behavioural function, with the observation that cap-dependent translation initiation results in the upregulation of polyribosomes in dendritic spine heads during memory consolidation in rats (7). Collectively, the decentralisation of mRNA expression and neuronal translation has emerged as a key mechanism through which plasticity may be restricted to distinct subcellular regions of the neuron. The post-transcriptional and translational regulatory systems which support protein synthesis under appropriate conditions, however, have remained poorly characterized.

Mounting evidence supports a major regulatory role for brain-enriched non-coding microRNA (miRNA) at the post-transcriptional and translational levels through target-specific repression of mRNA (8). Recently, miRNA have been observed to exhibit compartmentalised (9–12) and highly dynamic (13–15) expression within the neuron, implying miRNA support an important function in spatial and temporal restriction of neuronal gene expression. Despite a recent boom in miRNA studies, however, the mechanisms through which miRNA regulate neuronal gene expression are still poorly understood and the physiological effects of miRNA on neuronal plasticity have only been described for a small subset of these molecules. This review will explore and discuss current literature pertaining the role of miRNA in neuronal post-transcriptional and translational regulation, with particular focus on the mechanisms of mRNA repression involved, the subcellular distribution of neuronal miRNAs and their activity-dependent remodelling. In addition, the impacts of currently known miRNAs on neuronal structure and function associated with neuronal plasticity will also be explored to highlight their role as critical regulators of activity-dependent changes to neuronal function.

### MicroRNA biogenesis and target acquisition

miRNA are a family of ∼22nt non-coding RNAs initially characterized in 1993 as negative regulators of gene expression (16,17). Since this discovery, in excess of 3,700 unique mammalian miRNA have been discovered (18), which participate in a multiplicity of diverse functions including cellular differentiation, migration, proliferation, apoptosis, intracellular signalling and neurotransmission (19). As such, miRNA biogenesis, mechanisms of action and functional significance are currently the focus of intense research in various cellular contexts.

**Figure 1.**
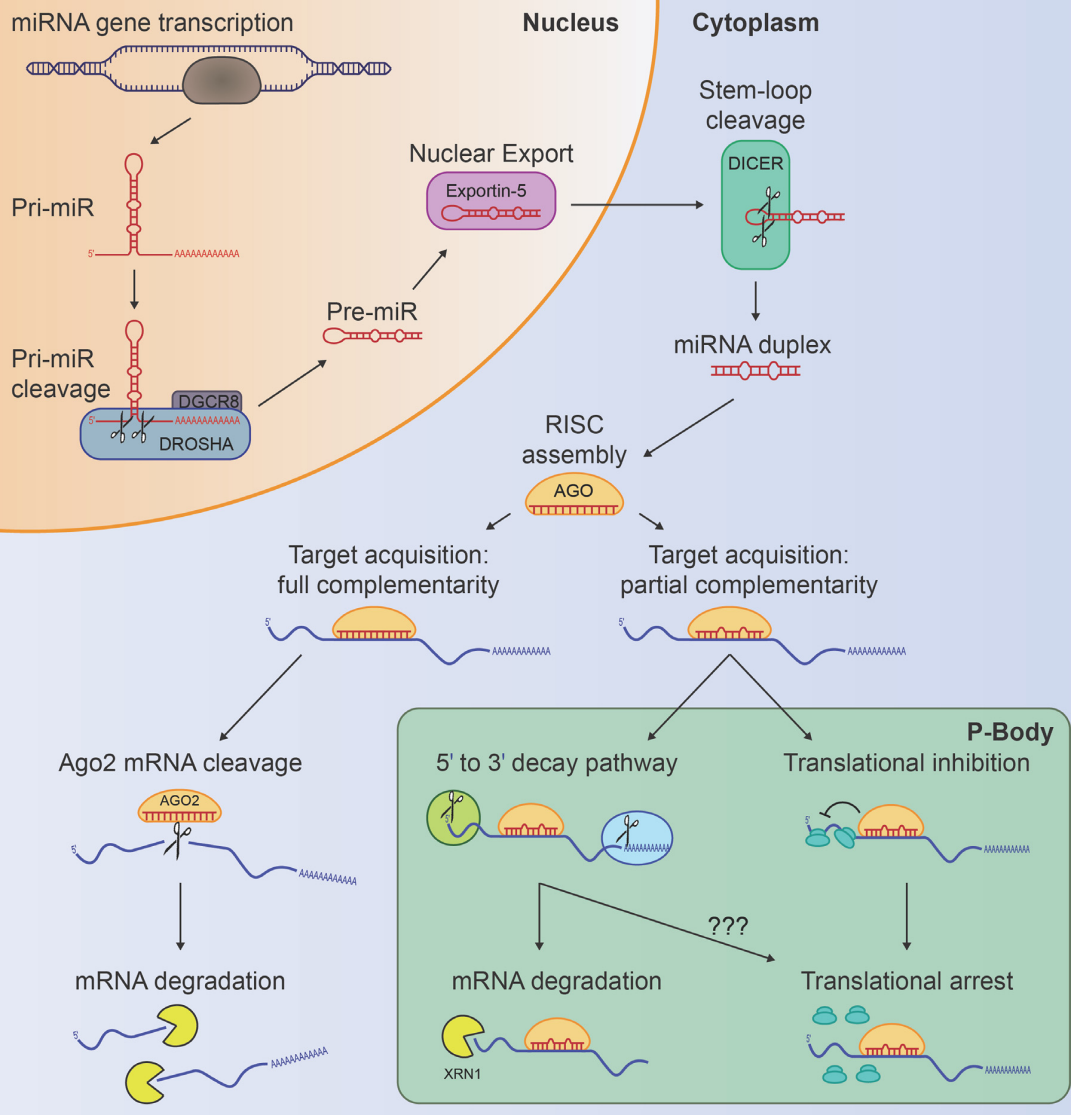
MicroRNA biogenesis and mechanisms of transcript repression. miRNA host genes are transcribed by RNA polymerase II, yielding primary miRNA (pri-miR) transcripts containing hairpin secondary structures (20). These ∼70nt hairpins, known as precursor miRNAs (pre-miRs), are liberated from pri-miRs by the Drosha-DGCR8 nuclear microprocessor complex (21) and then exported to the cytoplasm through nuclear pores upon association with Exportin-5 (22,23). In the cytoplasm, RNase III Dicer cleaves the hairpin loop to produce a double stranded miRNA duplex (24), from which one strand is loaded into an Argonaute (Ago) family protein (25–27). Full base complementarity between a miRNA associated with Ago2 and target mRNA results in direct endonucleolytic cleavage of the transcript by Ago2, followed by degradation (33). Partial miRNA-mRNA complementarity triggers either 5′ to 3′ mRNA decay—with potential for translational arrest—(30,31,37–41), or direct inhibition of mRNA translation (44–49,52,53,55,56), both of which are thought to be localized with subcellular P-bodies (62,63).

miRNA genes are typically transcribed by RNA polymerase II and processed by the Drosha-DGCR8 (DiGeorge syndrome chromosomal region 8) nuclear microprocessor complex to yield ∼70nt hairpin precursor miRNAs (pre-miRs) (20,21). Newly produced pre-miRs are then exported to the cytoplasm by Exportin-5 for further processing by RNase III Dicer, which digests the pre-miR hairpin loop (22–24). The resultant ∼22 bp miRNA duplex is loaded into an orthologue of the Argonaute (Ago) protein family (25,26), wherein a functional guide strand is selected and its antisense counterpart is degraded (27). These steps consequently lead to the formation of a mature RNA-induced silencing complex (RISC), which functions as a major post-transcriptional regulator of gene expression (Figure 1). As part of this complex, miRNA function to identify target mRNAs through complete complementary base pairing between the miRNA seed region (nucleotides 2–8) and miRNA recognition elements (MRE), encoded primarily within the mRNA 3′ UTR (8). The remainder of the miRNA sequence may then engage in either full or partial complementary binding with the target mRNA to dictate the mode of transcript repression. Since MREs are extensively distributed throughout protein-coding genes (28,29) and target acquisition requires recognition of short motifs, miRNA generally target multiple mRNAs, thereby establishing the basis for the exquisite co-ordination of gene networks as evidenced by the supported existence of over 366 000 miRNA-target interactions (18).

### MicroRNA fine-tune mRNA abundance

miRNA and the RISC have been traditionally characterized as mediators of transcript degradation, which has been observed in multiple eukaryotic systems (30,31). In plants, miRNA tend to exhibit perfect (or near perfect) sequence complementarity with target binding sites, which triggers direct mRNA cleavage by the RISC, followed by degradation (32). This process—known as RNA interference—may also be achieved in human cellular systems with Ago2-RISC (33); however, this is very uncommon (34,35) and generally requires exogenous introduction of synthetic small interfering RNAs (36).

The majority of human miRNA tend to engage in imperfect base-pairing with targets to promote degradation via the 5′ to 3′ mRNA decay pathway (30). In contrast to RNA interference, mRNAs committed to 5′ to 3′ degradation are initially subject to destabilisation via removal of the 3′ poly(A) tail and 5′ methylguanosine cap, prior to exonucleic digestion (30). Modification of mRNA within this pathway initially begins with deadenylation, whereby the RISC employs PAN2-PAN3 and CCR4-NOT deadenylase complexes to sequentially digest the poly(A) tail (37–39). The CCR4-NOT complex is then thought to directly stimulate decapping by recruiting the DCP2 decapping enzyme and associated co-factors to the 5′ region of the target mRNA, thereby providing a physical link between deadenylation and decapping (40). Cleavage of these structures ultimately exposes the transcript to digestion by XRN1 exonuclease (41), therein leading to decreased abundance of the target mRNA and therefore decreasing expression of the gene of interest.

### MicroRNA as regulators of mRNA translational competency

miRNA and the RISC have been increasingly implicated as regulators of translational activity, challenging the view that miRNA function as an irreversible ‘off’ switch for their target mRNA. In this regard, the 5′ to 3′ degradation pathway itself has become of recent focus, as miRNA-induced deadenylation and decapping inhibits pro-translational 5′ to 3′ mRNA interactions (42,43). Particular emphasis has been placed on miRNA-associated deadenylation, as removal of the poly(A) tail and associated binding proteins is thought to commit target mRNAs to translational arrest (44–46). There remains considerable debate, however, as to whether this process is functionally independent of, or simply precedes mRNA degradation (47). Stronger mechanistic evidence of miRNA acting as direct translational regulators has been observed with respect to interactions with eukaryotic initiation factors (eIF) bound to the 5′ extremity of target mRNAs, which are thought to be critical for ribosome assembly. A major finding in this context was reported by Mathonnet *et al*. (48), who showed that let-7 miRNAs inhibit translation initiation by interfering with eIF4F 5′ cap recognition in a mouse Krebs-2 ascites cell extract system. This observation was extended by the discovery of an eIF4E-like 5′ cap-binding motif within Ago2, suggesting the RISC may competitively bind the mRNA 5′ cap (49). Although this finding was initially disputed by Kinch *et al*. (50), further investigation revealed miRNA allosterically regulate the 5′ cap binding motif (51), thereby providing a target-specific dimension to direct miRNA-mediated translational arrest. In *Drosophila melanogaster* cell-free extracts, Ago1—which shares common features with mammalian Ago1–4—has also been shown to trigger dissociation of eIF4A from the mRNA 5′ cap (52), suggesting inhibition of translation initiation may act as an evolutionary conserved RISC function. Recent evidence has also revealed direct inhibition of ribosome assembly within the 5′ UTR may provide yet another dimension to RISC regulation of translational competency, which occurs through RISC recruitment of the translationally repressive eIF6 (53).

These observations together provide a compelling argument for the role of miRNA in the direct modulation of mRNA translational competency. Emerging studies exploiting the ribosome profiling method of global translational profiling (54), however, suggest that this aspect of miRNA function is considerably more complex than initially anticipated. For instance, a ribosome profiling investigation conducted by Guo *et al*. (31) demonstrated that exogenous introduction of miR-1 and miR-155 to HeLa cells primarily affected translation of target mRNAs through modulation of transcript abundance. Follow-up analysis of the same data by Cottrell *et al*. (55) contradicted these findings and suggested these miRNAs induced translational repression of mRNA targets, with the magnitude of repression relating to their translational efficiency status. Bazzini *et al*. (56) additionally incorporated ribosome profiling to show that miR-430 induces translational repression prior to target degradation in zebrafish embryos, again further obscuring the functional role of miRNA regulation of translation. Regardless, it is clear from these studies alone that this aspect of miRNA function is particularly complex and likely involves a series of both contextual and temporal cues to determine the mode of repression.

### microRNA and translational regulation in neurons

Are miRNA-mediated mRNA translational arrest and degradation intimately linked or distinctly separate functions in complex eukaryotic cells? This question is particularly important in neurobiology, where uncoupling of these functions would provide considerable advantage for regulating dynamic temporospatial gene expression patterns, particularly in distal regions of neurons, such as the axons and dendrites. Capacity for translational ‘recycling’ of mRNAs would substantially alleviate reliance on mRNA trafficking from the nucleus (57), and facilitate rapid modulation of localized protein expression in response to stimuli, such as synaptic transmission. Although the vast majority of studies suggest miRNA predominantly induce degradation of their targets and reduce the template for protein synthesis, emerging evidence suggests purely translational mechanisms are functionally significant in specific contexts.

A number of miRNAs participating in exclusive translational regulation of specific targets have been observed in recent work. For example, in pigment dispersing factor cell-pacemaker neurons, which control circadian rhythms in *D. melanogaster*, miR-92a was found to repress translation of SIRT2 (Sirtuin 2) mRNA without triggering degradation, causing these cells to exhibit decreased responsiveness to nicotine stimulation (58). A similar relationship was observed in mouse primary hippocampal neurons for miR-124, which directly binds to the 3′ UTR of PTPN1 (Protein tyrosine phosphatase non-receptor type 1) mRNA to specifically inhibit its translation, leading to deficits in AMPA (α-amino-3-hydroxy-5-methyl-4-isoxazolepropionic acid) receptor trafficking and consequently triggering disruption of synaptic transmission, plasticity and memory *in vivo* (59). miR-124 has also been observed to repress translation of AMPA receptor subunit GluA2 exclusively in the soma (60), indicating an overall robustness to miR-124 translational regulation in the modulation of neuronal excitability and plasticity. In addition, miR-182 also exhibits context-dependent repression of translation in the axon growth cone of *Xenopus laevis* retinal ganglion cells, wherein this miRNA silences localized translation of Cofilin-1 to inhibit axon guidance, a relationship which is abolished following pro-guidance Slit2 signalling (61).

Indirect support for the existence of purely translational mechanisms of miRNA function has also increased through studies focussing on subcellular granules known as P-bodies (processing bodies; also known as GW182 bodies due to their enrichment of Ago-associated GW182 proteins). P-bodies are thought to act as cytoplasmic foci of miRNA-mediated mRNA repression, which form upon the aggregation of 5′ to 3′ decay machinery, RISC components and translationally arrested mRNAs (62,63). Although mRNA degradation has indeed been observed within these subcellular granules (64,65), various paradigms of translational stress have further revealed that translationally arrested mRNAs may be stored within P-bodies and, in some cases, return to the pool of actively translated transcripts under appropriate conditions (66–69). These dynamics have particular functional significance with respect to gene expression associated with neuronal plasticity, as P-bodies have been observed to migrate to synapses via kinesin motor proteins (70) and dissociate in response to synaptic activity (71), perhaps releasing translationally arrested mRNAs from repression to fuel new protein synthesis. Although the exact mechanisms by which mRNA may regain translational competency after miRNA-mediated repression remain elusive, compartmentalised expression of effectors involved in mRNA capping and polyadenylation likely plays a key role. A primary example is the neuronally enriched poly(A) polymerase GLD-2, which is subject to cytoplasmic enrichment, enhances translation (72) and is required for induction of long term potentiation (*D. melanogaster*) and facilitation (*Aplysia*) (73). Taken together, these results suggest miRNA regulation of neuronal gene expression not only occurs via canonical and coupled repression of mRNA abundance and translation, but also may function through independent translational mechanisms. The nature of miRNA-mediated repression, however, appears to be highly context specific and is likely to be influenced by the spatial and temporal distribution of any given miRNA and its targets, as well as their functional significance.

### Subcellular compartmentalisation of neuronal microRNA

Given the capacity of miRNA to precisely regulate the stability and translation of mRNA, further investigation of their subcellular localisation in neurons provides important insight into their function. Early tissue profiling studies identified the CNS as a source of miRNA enrichment (74,75), with microarray (76) and deep sequencing (77) approaches establishing distinct profiles of expression within brain regions and neuronal subpopulations. Interestingly, spatially organised patterns of miRNA expression are also observable on a subcellular scale, where characteristic neuronal structures such as axons, dendrites and the soma exhibit localised expression of specific miRNA species (9–12). This discrete subcellular compartmentalisation of miRNA ensures local translational activity occurring in these regions is both spatially restricted and co-ordinated, particularly in distal axons and dendrites.

The development of synaptoneurosome preparations—vesicles containing the pre and post-synaptic compartments—proved a critical step in identifying mature miRNAs preferentially localising to the pre-synaptic bouton and dendritic spines. Using this technique, Lugli *et al*. (9) were able to identify as many as 37 distinct miRNA subject to enrichment within synaptoneurosomes derived from adult mouse forebrain neurons, compared to whole cell homogenate. These results were supported by a similar study conducted by Xu *et al* (11), who further identified that miRNA were released from the synaptosomal (pre-synaptic terminal only) fraction following application of a depolarizing Ca^2+^ stimulus. Subsets of miRNA identified in these studies—such as miR-134, miR-135 and miR-138—have since been characterised, with many undertaking crucial functional roles with respect to neuronal plasticity. Axon-specific miRNA have also recently been identified through neuronal cell culture methods optimised for axonal purification. For instance, utilizing a Campenot compartmentalised cell culture approach, Natera-Naranjo *et al* (12) discovered 130 miRNAs localised within the axon of sympathetic cervical ganglia neurons, of which 4—miR-15b, miR-16, miR-204 and miR-221—were enriched compared to cell bodies. Sasaki *et al*. (10) supported these results through an extensive quantitative real-time PCR study, which found in excess of 100 distinct miRNA species (out of 375 tested) were axonally enriched in cortical neurons, with six significantly enriched compared to cell bodies. Although the function of such miRNA is yet to be thoroughly investigated, recent studies suggest these miRNA likely participate in regulating axonal energy metabolism, growth and branching (78).

With so many unique miRNA localised to distal regions of the neuron, it is not surprising that the mechanisms of translocation have been subject to considerable discussion. One hypothesis proposed by Kosik in 2006 (79) suggested mature miRNAs and associated RISC proteins—including Ago, GW182, FMRP (Fragile X mental retardation protein) and MOV10 (Moloney leukemia virus 10 protein), amongst others (57)—may bind target mRNAs within the soma prior to translocation to distal regions of the neuron and direct RNA traffic. In such a system, cis-acting localisation elements encoded within the 3′ UTR of target mRNAs may determine the subcellular compartment in which these mRNA-miRNA-protein aggregates accumulate (57,80). Additional evidence, however, supports a model wherein precursor miRNAs are translocated to the site of need for processing into their mature, active forms. Evidence substantiating this hypothesis was recently observed in primary hippocampal and cortical neurons, where the dead-box helicase DHX36 was shown to regulate dendritic accumulation of pre-miR-134 through interaction with the pre-miR terminal loop (81). Interestingly, substitution of this loop structure with a pre-miR-150 loop sequence abolished dendritic accumulation of pre-miR-134, emphasising the importance of this motif in directing highly specific pre-miR distal translocation (81). Moreover, this accumulation of pre-miR-134 was shown to occur in an activity-dependent manner after BDNF (brain-derived neurotrophic factor) stimulation of NMDA (*N*-methyl-d-aspartate) receptors, resulting in dendritic outgrowth which bears strong functional relevance for neuronal miRNA localisation in activity-dependent neuronal plasticity (82). These findings support the previous discovery of precursor miRNAs and stimulus-dependent activation of processing enzyme Dicer within the post-synaptic density (PSD), axon and axon growth cone (13,83,84), which in itself implied that pre-miRNAs are processed into mature miRNAs at the site of need. Overall, these results support the idea that distal subcellular compartments of the neuron are capable of functioning as independent domains of gene expression with extensive capability for miRNA-mediated post-transcriptional regulation. Although evidence pertaining the role of miRNA spatial distribution in neurological disorders remains limited, the activity-sensitive nature of miRNA trafficking alone suggests this may be functionally significant for disorders characterised by aberrant responses to synaptic activity.

### MicroRNA expression is remodelled by neuronal activity

**Figure 2.**
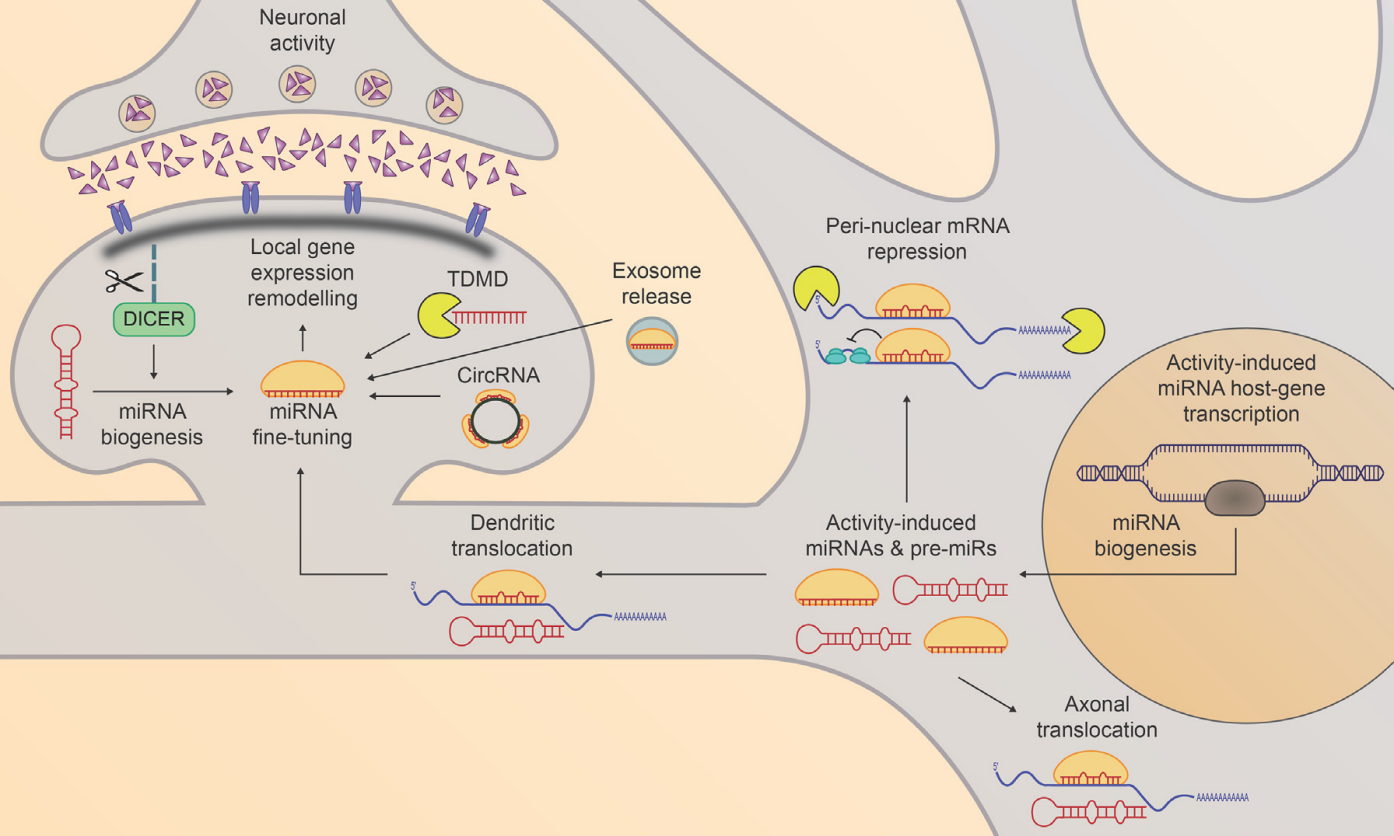
Mechanisms of activity-associated modulation of neuronal microRNA. Synaptic activity triggers a cascade of reactions leading to remodelling of neuronal miRNA expression. In close proximity to the synapse, Calpain-I cleaves Dicer from the post-synaptic density, allowing localised processing of precursor miRNAs into mature miRNAs (13,89). In concert, specific miRNAs tagged for activity-induced suppression are subjected to sponging by circular RNA (91–96), target RNA-directed miRNA degradation (99–101) or are ejected from the neuron in exosomes (97,98). Excitatory signalling towards the soma results in activity-induced miRNA host gene transcription and processing (85–88), modifying miRNA expression in peri-nuclear regions which leads to modulation of gene expression in the soma. These miRNA (57,79,80) or their precursors (81,82) may additionally be shipped to dendrites or the axon for regulation of distal gene expression.

Activity-induced mRNA translation is thought to undergo rapid, spatially controlled changes to provide both localized and global changes to the neuronal proteome. To establish and co-ordinate appropriate profiles of gene expression in response to synaptic stimuli, it has become increasingly clear that miRNA themselves are subject to activity-associated fine-tuning (Figure 2). This concept is particularly evident in paradigms of long-lasting neuronal plasticity such as LTP and LTD, where high-throughput analyses have identified subsets of miRNA thought to play critical regulatory roles (14,15). Although these patterns of miRNA expression are becoming increasingly clear, the overall basis of activity-associated miRNA expression is complex and thought to involve a number of distinct mechanisms important for directing neuronal responses to excitation.

Stimulus-dependent transcription of miRNA genes has been observed as a traditional means by which processing of mature miRNAs may be induced in response to neuronal activity. A major example is miR-132, which is upregulated following CREB-dependent (cAMP response element binding protein) transcription of its host gene after pharmacological stimulation of hippocampal neurons (85,86). This robust increase in miR-132 expression coincides with a key role in promoting neuronal signalling and morphology associated with enhancement of LTP (85,87), indicating this regulatory loop may play a key role in dictating neuronal plasticity. Transcriptional regulation of neuronal miRNA is also thought to extend to entire miRNA genomic clusters to achieve widespread remodelling of post-transcriptional regulation. For instance, Fiore *et al*. (88) demonstrated the Mef2 (Myocyte enhancer factor-2) transcription factor mediates increased production of the miR-379–410 cluster—which contains in excess of 50 miRNA species—in cultured mouse cortical neurons after BDNF or KCl treatment. This interaction is thought to mediate activity-dependent dendritic remodelling through upregulation of important neuronal miRNAs, such as miR-134 (88).

Increasing emphasis has been placed on elucidating the nature of activity-associated synthesis of mature miRNAs in distal neuronal compartments, as miRNA remodelling in these regions is thought to be fundamentally important in the regulation of localized mRNA translation. This phenomenon was first detected in NMDA-treated mouse hippocampal slices by Lugli *et al*. (13), who identified an NMDA-induced stimulation of Dicer at the PSD. In this model, activation of Calpain-I by acute rises in post-synaptic intracellular calcium was observed to cleave Dicer from the PSD and consequently increase Dicer activity, suggesting a direct link between neuronal excitation and miRNA production in close proximity to activated synapses. This spatially restricted, activity-associated miRNA production has since been supported via single synapse stimulation, whereby uncaging of a glutamate stimulus in the vicinity of hippocampal dendrites induced discretely localized processing of a pre-miR-181a probe (89). These studies together suggest that activity-dependent miRNA induction may be regulated by both novel miRNA host gene transcription as well as localized synthesis at the site of need to support rapid changes in distal gene expression.

Distinct profiles of miRNA downregulation have also been observed in response to neuronal activity (14,15), however the mechanisms underpinning this remodelling have only recently been subject to increased focus. Since miRNA are thought to be largely stable relative to other classes of RNA due to their association with Ago proteins (90), reduction of miRNA functional capacity is thought to be primarily achieved via sequestration of specific miRNA, rather than degradation. A class of novel, exonuclease-resistant circular non-coding RNA—known as circRNA—may provide this layer of regulation through competitive binding of miRNA species, acting as makeshift miRNA sponges or competing endogenous RNA (ceRNA) (91–93). Prominent examples in the literature include circRNA CDR1as (91,92) and circHIPK3 (93), both of which have been shown to contain multiple seed-region binding sites utilized for potent downregulation of target miRNAs including miR-7, miR-124 and miR-588. CDR1as in particular appears to participate in a complex miRNA regulatory loop, which involves potent sponging of miR-7, but also its own endonucleolytic degradation upon binding of miR-671 to a single, highly complementary miRNA recognition element (94). Profiling studies have revealed that circRNA exhibit neuronal enrichment—particularly within dendrites (95)—and respond dynamically to bicuculine-induced neuronal activity (95), implying an important functional role in activity-associated regulation of miRNA. While their overall impacts on neuronal function remain obscure, loss of CDR1as has been shown to induce dysfunction of excitatory synaptic transmission in mice (94), while upregulation of the circular miR-615 sponge cZNF609 was recently found to contribute to degeneration of retinal neurons (96), suggesting disruption of this miRNA regulatory mechanism results in major disturbances to neuronal function.

Another mechanism by which subsets of miRNA may be rapidly downregulated is expulsion via exosome release, which has recently been observed in neuronally differentiated SH-SY5Y cells subject to membrane depolarization (97). Microarray analysis revealed these Map1b-enriched exosomes contained miR-638, -149*, -4281 and let-7e, which were shown to be downregulated in the neurite fraction (97). Since exosomes are generally stable within the extracellular environment, ejection of miRNAs via this mechanism may additionally serve as a form of auxillary communication with other neurons and glial cells. This potential for cross-talk was recently supported by findings in dorsal root ganglion neurons after exposure to capsaicin (*in vitro*) or spared nerve injury (*in vivo*) (98). Interestingly, the affected neurons were shown to release exosomes containing miR-21-5p from the soma which were phagocytosed by macrophages, subsequently resulting in the polarization of macrophages towards pro-inflammatory phenotypes (98). Therein lies potential for miRNA-containing vesicles to act as a vehicle of neuronal paracrine communication after excitation.

Target RNA-directed miRNA degradation (TDMD) has also recently emerged as a novel mechanism of neuronal miRNA downregulation, whereby extensive complementarity with a target RNA leads to miRNA destabilisation and decay through 3′ trimming or the untemplated addition of A residues (99). Complementary base pairing in this context is thought to include central mismatches to preclude the chance of miRNA-mediated endonucleolytic cleavage (99). In primary rat hippocampal neurons and mouse cerebellar granule neurons, introduction of exogenous constructs complementary to a subset of neuronal miRNA revealed TDMD exhibits distinct potency in reducing levels of miR-124, miR-128, miR-132 and miR-138, all of which have previously been identified as highly important neuronal miRNAs (99). More recently, an endogenous long non-coding RNA known as Cyrano has additionally been shown to repress miR-7 expression (100). Surprisingly, loss of Cyrano and subsequent accumulation of miR-7 resulted in miR-671-directed loss of circRNA CDR1as, suggesting increased miRNA sponging may potentiate circRNA degradation (100). Although investigation of this mechanism in neurons is still in its infancy, as a whole TDMD appears to bear critical importance for synaptic function involved in neurobehavioural control. Specifically, the long non-coding RNA Libra in zebrafish and its mammalian protein-coding ancestor NREP1 (neuronal regeneration related protein) in mice was found to regulate multiple behavioural traits in both species through modulation of miR-29 (101), which itself has been previously implicated as a key regulator of neuronal viability in the hippocampus and cerebellum (102).

### microRNA dynamically regulate neuronal excitability

Since miRNA expression is intrinsically linked with neuronal activity, it is perhaps not surprising that many miRNA have been implicated in the dynamic regulation of neuronal excitability, bearing significance for both short and long-term forms of neuronal plasticity. Although the currently described miRNAs in this context are likely to regulate entire gene expression networks with relevance to neuronal activity, the majority of current studies investigating the mechanisms and functional roles of these miRNA have predominantly focussed on detailing individual miRNA-target pairings with direct functional consequences. As the expression of neurotransmitter receptors and ion channels is especially important for the maintenance or regulation of neuronal excitability, recent studies have placed emphasis on identifying critical miRNA-mRNA interactions involved in regulation of such factors. miRNA-mediated repression of NMDA and AMPA class glutamate receptors in particular has formed key focus due to their role in the modulation of excitability associated with LTP and LTD (103,104), and as such, many currently described miRNA act to antagonise increases in excitatory capacity. An important example of a miRNA engaging in such an interaction is the Schizophrenia-associated miR-137, which directly represses the GluA1 AMPA receptor subunit to enhance synaptic silencing and mGluR-dependent LTD (105). Sponging of this miRNA consequently inhibits induction of chemically induced LTD in the CA1 hippocampus, reflected in a loss of long-term decline in evoked excitatory post-synaptic currents (105). A similar phenotypic outcome is achieved by miR-92a (106) and miR-501-3p (107), which also negatively regulate GluA1 expression, and miR-125b, which decreases expression of the NR2A NMDA receptor subunit to achieve synaptic silencing (108). miR-135, on the other hand, acts through an indirect regulatory mechanism following upregulation during NMDA-evoked LTD. This miRNA was recently shown by Hu *et al*. (109) to decrease expression of SNARE-interacting proteins Complexin-1 and -2, leading to a net decrease in GluA1 exocytosis to the post-synaptic membrane.

These findings together emphasise a distinct redundancy of miRNA function in achieving a phenotypic outcome, an important aspect of miRNA dynamics to consider when designing studies focussing on functional consequences of specific miRNA-mRNA interactions. It is also important to acknowledge that the discussed interactions are relatively simple cases to reconcile with changes in neuronal excitation, whereas many other miRNA are predicted to be involved through complex regulatory systems. For instance, Sosanya *et al*. (110) recently identified miR-129 as a positive regulator of neuronal excitability in rat hippocampal neurons, which functions via translational repression of Kv1.1 voltage-gated potassium ion channels in dendrites. Interestingly, this interaction is capitulated by binding of the translational regulator HuD in response to a reduction of pro-translational mTORC1 signalling (110), suggesting neuronal excitability is decreased in response to a decline in overall translational activity. Another distinctly complex miRNA in this context is miR-132, which promotes neuronal excitability in rat cortical neurons after BDNF-induced upregulation via the MAPK/ERK pathway. This miRNA leads to increased expression of NR2A, NR2B and GluR1 glutamate receptor subunits (87), though the exact mechanism by which miR-132 upregulates these targets remains elusive and is likely to involve layers of upstream regulation.

### MicroRNA modify neuronal structural plasticity

Neuronal cytoarchitecture plays a major role in enhancing or suppressing synaptic communication within neuronal circuits, and hence, modifications to neuronal morphology occur in a profoundly dynamic manner. Changes to dendritic structure in particular have been shown to bear crucial importance in long-lasting forms of plasticity such as LTP and LTD, wherein increases in the number and size of dendrites and spines is thought to potentiate post-synaptic neurons, while opposite effects are thought dampen responses to synaptic input (111,112). Naturally, many miRNA have been implicated in the regulation of underlying patterns of gene expression associated with neuronal structural modifications, and often, exogenous modulation of specific miRNA species results in distinctly abnormal morphological phenotypes.

miR-134 is widely regarded as the first major miRNA described in this context, which was initially observed in a comprehensive functional study by Schratt and colleagues in 2006 (113). When overexpressed in cultured rat hippocampal neurons, miR-134 was shown to induce a distinct decrease in dendritic spine size through synapto-dendritic repression of Limk1 (Lim-domain-containing protein kinase 1), a kinase associated with regulation of actin filament dynamics (113). A subsequent study by Fiore *et al* (114) further identified that miR-134 inhibition of RNA binding protein and translational repressor Pum2 (Pumilio RNA binding family member 2) decreases activity-dependent dendritogenesis and promotes homeostatic synapse pruning, illustrating an overall functional robustness to miR-134-mediated depression of synaptic signalling. Many other miRNA-target interactions have since been shown to play a key role in negative regulation of dendritic structure, including miR-29 (Arpc3) (115), miR-125b (NR2A) (108), miR-135 (Complexin-1/-2) (109) and miR-138 (APT1) (116), all of which reduce dendritic spine size, miR-125a (PSD-95) (117) and miR-485 (SV2A) (118), which serve to decrease overall dendritic spine density, and miR-137 (Mind bomb-1) (119), a negative regulator of dendritic spine complexity and length. The overall enrichment of distinct miRNAs in repression of these structural features suggests the involved miRNAs may act as a molecular restraint for neuronal structural plasticity, likely functioning to antagonize over-potentiation of synaptic circuits.

Downregulation of the aforementioned miRNAs is thought to contribute to morphological potentiation of dendrites and dendritic spines, effectively ‘releasing the brakes’ on expression of major structural regulators. Although this is yet to be thoroughly investigated for the examples given, emerging evidence suggests this concept may hold functional merit for other miRNA. Specifically, downregulation of miR-26a and miR-384-5p 90 minutes after LTP induction in the mouse hippocampus has been shown to release RS6K3 (ribosomal S6 kinase 3)—a protein kinase which regulates translational control pathways—from functional repression (120). The resultant signalling is required for prolonged LTP maintenance through modification of dendritic morphology (120). Other miRNA appear to positively correlate with the enhancement of structural plasticity through various interactions. For example, activity-dependent induction of miR-132 decreases translation of p250GAP in rat hippocampal neurons, leading to dendritic growth, branching and expression of mushroom-shaped spines (85). In concert with this role, miR-132 expression additionally enhances dendritic growth, arborisation, spine density and synaptic integration in newborn neurons of the adult hippocampus (121), dentate gyrus (122) and neonatal olfactory bulb (123), suggesting an overall critical function in the development and maintenance of synaptic morphology. miR-188 also acts as a positive regulator of synaptic structure associated with potentiation through decreasing expression of Neuropilin-2, a neuronal semaphorin receptor associated with decreased dendritic spine density and synaptic structure (124).

### Neurobehavioural effects of microRNA function

Patterns of miRNA expression, and the resultant changes to gene expression evidently play a major role in maintaining and dynamically modulating neural plasticity. Many of the resultant modifications to neuronal excitability and morphology have been directly observed in models of synaptic plasticity, the hypothesised biological mechanism behind the phenomena of learning and memory (125). Accordingly, disruption of miRNA function *in vivo* in brain regions such as the hippocampus has been shown strongly influence performance in behavioural paradigms associated with memory. This concept was demonstrated in a study by Konopka *et al* (126), who developed a conditional forebrain *Dicer1* knockout mouse to investigate the effects of total miRNA ablation. Subsequent remodelling of dendritic spine structure and increased expression of synapse-associated proteins were, in part, thought to contribute to an enhancement of spatial and fear-associated memory after Morris water maze and fear conditioning paradigms (126). These results have since been supported in a similar model by Fiorenza *et al*. (127), whereby increased excitability of hippocampal CA1 pyramidal neurons was reflected in enhanced contextual fear conditioning.

Several specific miRNA species have been implicated in the regulation of neurocognitive functions associated with synaptic plasticity. These studies suggest that aberrant regulation of a subset of genes may result in overall abnormal neurobehavioural phenotypes. For example, overexpression of miR-134 in the CA1 region of the hippocampus results in contextual fear learning deficits in mice, indicative of the role miR-134 plays in negative regulation of dendritic spine size (128). Similar outcomes have been observed for miR-137 when overexpressed in the mouse dentate gyrus (129). Although the behavioural effects of these miRNA are easily reconciled with their observed roles in regulating synaptic signalling and morphology, linking these aspects of miRNA function is not always so simple. This is particularly true of miR-132, which impairs novel object recognition memory when overexpressed in the CA1 hippocampus despite being shown to increase dendritic spine density in the same paradigm (130). Disparity between these observations and previous reports of miR-132 potentiating synaptic transmission (85,87,121–123) suggest miR-132 function is exceptionally complex. Other miRNA, such as miR-92 miR-128b and miR-182, amongst others, appear to exhibit expression regulated by neurocognitive experiences (131–133), implying an important underlying functional role. As a result, these miRNA are prime candidates for future characterisation in terms of targets and impacts on neuronal plasticity.

### Future perspectives and challenges

From the discussed evidence, it is clear that miRNA regulation of gene expression has particularly distinct complexity and biological significance in the neuronal context. Although investigating the interactions and physiological impacts associated with individual miRNA has been an essential aspect of research effort, the mechanistic details associated with miRNA expression and the discrete decision-making processes involved, is of key importance to fully appreciate how these molecules operate. One dimension of miRNA function which will likely form key focus in coming years with regards to the neuron is the modulation of mRNA translational competency, which presents an especially interesting and seemingly logical system by which subcellular neuronal compartments such as dendritic spines could express genes in a semi-autonomous manner. Recent reports of miRNA acting as translational regulators supports the existence of such a system (48,49,51,52,58–60), and likewise, migration of mRNAs from P-bodies to polysomes adds further weight to this hypothesis (68,69). However, the extent by which miRNA-mediated mRNA degradation and translational repression may be uncoupled requires further investigation, which will likely benefit from new high-throughput methods, such as ribosome profiling (54) and others (Table 1) to capture the global effects of miRNA on translation. Additional attention should also be placed on integrating miRNA function with mechanisms of neuronal mRNA trafficking, considering mounting evidence suggests there is crosstalk and structural similarity between neuronal messenger ribonucleoprotein granules—aggregates of mRNA and RNA binding proteins thought to be the vehicle of neuronal mRNA transport—and P-bodies (71,134). These observations suggest a degree of functional overlap exists between delivery of mRNA to subcellular compartments and repression by miRNA.

**Table 1.** High-throughput sequencing strategies relevant to investigation of microRNA post-transcriptional function

Sequencing strategy	Feature analysed	Applications	Reference(s)
mRNA-Seq	Poly(A) RNA expression	Identification of mRNAs committed to degradation or upregulation with respect to miRNA expression.	Nagalakshmi *et al*. (136).
Small RNA-Seq	Small RNA expression	High-throughput, global quantification of mature miRNA.	Lu *et al.* (137); Hafner *et al.* (138).
Ribosome profiling (Ribo-Seq)	Active mRNA translation	Nucleotide resolution of actively translated mRNAs. May be integrated with mRNA expression to elucidate miRNA targets selectively regulated at the level of translation, particularly in the context of synaptic miRNA function.	Ingolia *et al.* (54).
Poly(A) tail sequencing (PAT-Seq)	mRNA poly(A) tail length mRNA 3′UTR usage	Resolves changes in mRNA poly(A) tail length which may be correlated with miRNA-induced 5′ to 3′ degradation. Additionally provides high-resolution analysis of alternate 3′ UTR sequences, which modifies abundance of miRNA binding sites.	Harrison *et al.* (139).
Cap analysis gene expression sequencing (CAGE-Seq)	mRNA 5′ extremity	Identification of miRNA binding sites housed within the mRNA 5′ UTR, which may have functional relevance for translational regulation.	Shiraki *et al.* (140); Takahashi *et al.*, 2012 (141).
RNA immunoprecipitation sequencing (RIP-Seq)	Protein-associated RNA	May be utilized to identify mRNAs complexed with proteins known to associate with microRNA and regulate mRNA stability/translation (e.g. Ago2, FMRP, MOV10)	Keene *et al.* (142).
Cross-linking, ligation and sequencing of hybrids (CLASH-Seq)	mRNA-microRNA interactions	Direct quantitative analysis of mRNA-microRNA pairings.	Helwak *et al.* (143).
Mammalian native elongating transcript sequencing (mNET-Seq)	Nascent transcription	Integration with mRNA expression data for dissection of mRNAs subjected to upregulation via transcriptional activity and mRNAs upregulated through release from miRNA repression.	Nojima *et al.* (144).
Parallel analysis of RNA ends (PARE-Seq)	Degrading RNA	Identification of microRNA targets subjected to degradation.	German *et al.* (145).
m6A-methylated RNA sequencing (m6A-Seq)	m6A RNA methylation	Transcriptomic profiling of mRNA m6A methylation events, thought to have functional relevance for post-transcriptional miRNA regulation of mRNA.	Meyer *et al*. (146).
Circular RNA-Seq (Circ-Seq)	Expression of circular, RNAse R resistant RNA	Analysis and identification of circRNA with potential to function as microRNA sponges.	Memczak *et al*. (92); Nair *et al.* (147).

Our understanding of miRNA in the regulation of neural plasticity is slowly emerging and likely to expand further in coming years as new miRNA are implicated and characterized. While over 3700 mammalian miRNA are currently supported by evidence (18), and a large proportion are expressed in the brain, the function of most of these is currently unknown. It is likely that many of these are involved in regulating neuronal plasticity through both direct regulation of key effectors and upstream regulation of entire gene expression networks. This gives further impetus for functional studies of individual miRNA, particularly those that can reconcile overall changes in the microscopic neuronal phenotype with the associated behavioural phenotype, especially in psychiatric disorders associated with deficits in neural plasticity that have been associated with specific miRNA, such as schizophrenia, major depressive disorder, bipolar disorder, autism spectrum disorders and addiction (135). Ultimately, these molecules may have clinical significance in novel therapeutic strategies that can enhance or normalize their dysregulation in the brain.

## CONCLUSIONS

Post-transcriptional gene regulation is vital to organizing the intricate patterns of intracellular protein synthesis that supports complex cellular morphology, cellular networks and systems. The vertebrate brain has optimized these systems by incorporating small non-coding RNA as the universal guide to mediate the highest specificity of interactions at the lowest biological cost. In this review we have summarized what is known about this remarkable system, as there is increasing evidence that post-transcriptional regulation of gene expression is critical for neuronal plasticity and its neurobehavioural manifestations. We are currently just scratching the surface of this mechanism, and further investigation of miRNA in particular is likely to reveal significant new insights into the complexity of neuronal gene expression and function.
